# Morphological and biochemical responses to water stress in *Solanum pimpinellifolium* and *S. lycopersicum var. cerasiforme* accessions

**DOI:** 10.1007/s12298-026-01705-7

**Published:** 2026-01-20

**Authors:** Aylin Kabaş, Oussama Antar, Hayri Üstün, Santiago Vilanova, Oscar Vicente, Jaime Prohens

**Affiliations:** 1 Instituto de Conservación y Mejora de la Agrodiversidad Valenciana, Universitat Politècnica de València, Camino de Vera 14, 46022 Valencia, Spain; 2 Department of Organic Farming, Manavgat Vocational School, Akdeniz University, 07070 Antalya, Turkey; 3 Laboratoire de Recherche en Sciences Végétales — Génomique et Biotechnologie des Fruits — UMR 5546, Université de Toulouse, CNRS, UPS, Toulouse INP, Toulouse, France

**Keywords:** Tomato, Water stress, Proline, Osmotic adjustment, Growth parameters

## Abstract

Drought is a critical abiotic stress that negatively affects the growth and productivity of tomato (*Solanum lycopersicum* L.). In this study, we assessed the morphological and biochemical responses of plants from eight accessions of tomato relatives, four of *Solanum pimpinellifolium* (SP) and four of *S. lycopersicum* var. *cerasiforme* (SLC), under control (100% field capacity, FC) and water stress (40% FC) conditions. Growth parameters, including stem, leaf, and root morphology, biomass, water use efficiency (WUE), photosynthetic pigments (chlorophyll a, chlorophyll b, and carotenoids), and osmolytes (proline and total soluble sugars) were assessed. Thedifferences between accessions, treatments, and their interactions were assessed. In drought conditions, the two accessions, ECU689 (SP) and ECU1009 (SLC), were identified as the most drought-tolerant ones. The two genotypes hardly showed a decrease in root water content, root fresh weight, and aerial biomass, as well as small alterations of leaf morphology. All these features point to their better performance of growth and physiological function under stress. Although proline levels increased under water stress in all accessions, the rise was markedly lower in ECU689 and ECU1009, suggesting that these accessions may utilize alternative adaptive mechanisms, such as root resilience and osmotic regulation. PT210 (SLC) demonstrated strong osmotic adjustment, increasing total soluble sugars by 183.2% (86.15 mg eq. glucose g^−1^ DW under stress). Photosynthetic pigments remained stable across treatments, indicating that the water stress applied was moderate. Principal component analysis (PCA) revealed a clear separation between the control and water stress groups; however, no clear separation was observed between SP and SLC accessions. Among the traits contributing to this differentiation, proline displayed significant positive correlations with photosynthetic pigments under water stress conditions, but not under control conditions. Overall, these findings highlight ECU689 and ECU1009 as promising donor candidates for breeding drought-tolerant tomato varieties, due to their ability to maintain stable root traits and biochemical balance under water stress. In contrast, among the other accessions, ECU1385 (SP) and PI487625 (SLC) were found to be more sensitive to stress. Incorporating these tolerant accessions into tomato breeding programs may facilitate the development of resilient tomato cultivars and rootstocks for drought-prone environments.

**Supplementary Information:**

The online version contains supplementary material available at 10.1007/s12298-026-01705-7.

## Introduction

Climate change is the most significant factor impacting agricultural productivity worldwide. Drought spells, which are predicted to increase in many regions as a consequence of climate change (IPCC 2021), are an important abiotic stress factor that limits production and causes severe reductions in yield (Cakmak 2005; Zhang et al. 2020). Drought is a consequence of a deficit of water in the soil, leading to various physiological and metabolic disorders in plants (Huang et al. 2019). Drought stress induces plant dehydration, leading to osmotic stress that damages cells and tissues, ultimately causing yield and quality losses (Wang et al. 2003). Plants have developed a series of mechanisms, including morphological, biochemical, physiological, and molecular adaptations, to cope with this stress condition. These strategies used by plants to withstand drought stress include shortening their life cycle (Chaves et al. 2003), improving the root system, reducing leaf area, and limiting transpiration (Xu et al. 2010). Some plants can handle drought stress by making their cell walls more flexible and better adapted to changes in osmotic pressure (Zhang et al. 2022). To survive under drought conditions, plants can alter their metabolic pathways, such as by enhancing antioxidant metabolism (Kapoor et al. 2020). In this regard, phenolic metabolism is particularly noteworthy, as it encompasses a diverse array of biological functions that may play a critical role in modulating drought stress tolerance (Sanchez-Rodriguez et al. 2010). Therefore, knowledge of the biochemical responses to drought is critical to comprehensively understand plant resistance mechanisms to water stress (Anjum et al. 2011).

Tomato (*Solanum lycopersicum* L.) is one of the most important crops in the world and is sensitive to drought (Foolad et al. 2003). Many studies have been conducted to characterize cultivated tomato susceptibility to different levels of water stress and to understand the molecular and physiological processes of drought tolerance in tomatoes (Foolad 2007; George et al. 2013; Jangid and Dwivedi 2016; Klunklin and Savage 2017; Martínez-Cuenca et al. 2020; Nahar and Gretzmacher 2011; Rahman et al. 2004). In this way, it has been found that drought causes adverse effects such as the reduction of shoot, root, fruit size and yield during the development of tomatoes (Nuruddin et al. 2003; Zhou et al. 2019).

Photosynthetic pigments, osmolytes, and oxidative stress markers are widely used biochemical markers to assess plant stress responses and stress tolerance mechanisms (Andrade et al. 2016; Zahedi et al. 2022). The reduction of photosynthetic pigment contents during drought stress in tomato is closely associated with diminished photosynthetic efficacy (Martínez-Cuenca et al. 2020). These pigments encompass carotenoids, chlorophyll a, and chlorophyll b, which are crucial for light absorption and energy transformation (Sharma et al. 2020). In response to water scarcity, osmolytes such as proline and soluble sugars accumulate, helping osmotic adjustment and protecting cellular structures from dehydration (Ashraf and Foolad 2007). Additionally, oxidative stress markers, particularly malondialdehyde (MDA), reflect lipid peroxidation caused by the excessive generation of reactive oxygen species (ROS), making them reliable indicators of oxidative damage under drought and other stressful conditions (Zhang et al. 2021).

Some wild tomato species such as *S. peruvianum* L., *S. chilense* (Dunal) Reiche, *S. pennellii* Correll, and *S. pimpinellifolium* L. have been reported as tolerant to drought stress (Foolad et al. 2003; Dariva et al. 2021; Tapia et al. 2016; Du et al. 2025; Rose et al. 2025). Also, some materials of the weedy tomato *S. lycopersicum* var. *cerasiforme* have been found to display tolerance to drought (Martínez-Cuenca et al. 2020). This suggests that tomato wild and weedy relatives can play a major role in the introgression of tolerance to drought into cultivated tomato. Here, we evaluate four accessions each of *S. pimpinellifolium* and *S. lycopersicum* var. *cerasiforme*, which are the parents of a multi-parent advanced generation intercross (MAGIC) population (Arrones et al. 2024), for tolerance to drought. In a previous study, where photosynthetic and morphological traits were evaluated, Martínez-Cuenca et al. (2020) determined that significant variation existed among these eight accessions for their response to water deficit and salt stress. However, no study has been performed on biochemical traits and root development related to drought tolerance. The aim of this study is to assess the impact of water deficit stress on the aerial and root morphological traits and biochemical characteristics (such as photosynthetic pigments, osmolyte concentrations, and oxidative stress levels) of plants of these eight accessions. Although the number of accessions evaluated was limited to eight, they represent the complete set of founders of the *S. pimpinellifolium* × *S. lycopersicum* var. *cerasiforme* MAGIC population (Arrones et al. 2024), selected for their wide genetic and phenotypic diversity and contrasting responses to abiotic stress. This subset thus provides a representative framework for assessing parental variation in morphological and biochemical responses to drought prior to large-scale population analyses. The results obtained will therefore contribute not only to understanding the biochemical and morphological differences between *S. pimpinellifolium* and *S. lycopersicum* var. *cerasiforme* germplasm, but also to identifying relevant parameters for evaluating drought tolerance in the MAGIC population derived from them.

## Material and methods

### Plant material

In this study, four accessions of *S. pimpinellifolium* (ECU1385, ECU689, Mex116, and LA2251) and four accessions of *S. lycopersicum* var. *cerasiforme* (PT210, T11, PI487625 and ECU1009) selected for their genetic and phenotypic diversity as well as their different origins, were used as plant material (Fig. S1). The *S. pimpinellifolium* accessions originate from regions spanning Manabí province in Ecuador to the northern and southern coastal areas of Peru, whereas *S. lycopersicum* var. *cerasiforme* accessions come from diverse locations, including the Sinaloa desert in Mexico and San Martín in Peru (Martínez-Cuenca et al. 2020). These accessions are the parents of a MAGIC population (Arrones et al. 2024; Martínez-Cuenca et al. 2020).

### Plant cultivation and treatments

The tomato seeds of the selected accessions were germinated in Petri dishes (9.0 × 2.5 cm) on a layer of hydrophilic cotton covered by filter paper. After ten days, the seedlings were transferred to a tray in a climatic chamber under fluorescent light with a 14 h light/10 h dark photoperiod at 25 °C. Seedlings were transplanted to pots (15 cm diameter × 14 cm height) filled with commercial peat (Humin substrate N3, Klasmann-Deilmann, Germany), with five replicates per treatment (control and water stress) and accession (ten plants in total from each parent) 25 days after sowing (DAS). Plants were placed in a greenhouse at the Universitat Politècnica de València with temperature control, ranging between 18 and 28 °C. Plants were distributed within the greenhouse according to a fully randomized design. The water stress treatment was initiated one week after the plants were transplanted into the greenhouse. The gravimetric method was used to perform the water stress treatment (Schulker 2023). This method is suitable for estimating differences between accessions in pot-culture studies (Shao et al. 2008). To prevent water loss by evaporation, after the pots were fully irrigated to the point of saturation, they were covered with aluminium foil for 48 h. Two irrigation regimes, control (C) and water stress (WS), were applied to the plants. The control plants were watered to field capacity (FC), whereas for the WS treatment, they were watered to 40% FC. The plants were irrigated twice a week. The pots were randomly rearranged each week during the greenhouse experiment to ensure uniform growth conditions. The experiment was concluded 30 days after the initiation of the stress treatment.

### Morphological evaluation

Root branching frequency (RBF), root average diameter (RAD), and root length (RL) were evaluated on roots gently cleaned at the end of the experiment using RhizoVision Explorer software to assess the effects of the treatments (Seethepalli and York 2020). Stem length (SL) was measured with a scale and leaf number (LN) was counted, while leaf length (LL) and leaf width (LW) were measured on the third leaf from the bottom of each plant. ImageJ processing software was used for the measurements (Ferreira and Rasband 2012). Leaf fresh weight (LFW) and water content (LWC), root fresh weight (RFW) and water content (RWC), and stem fresh weight (SFW) and water content (SWC) were measured for all plants. Plant leaf samples used to determine biochemical parameters were flash-frozen in liquid N_2_ and stored at -80 °C. All plant materials (roots, stems, and leaves) were collected separately and weighed (FW), dried in the oven at 65 °C for 48–72 h until constant weight, and then reweighed (DW) to calculate the water content as a percentage of each sample using the following formula: WC = [(FW − DW)/FW] × 100. The WUE was assessed using the water applied during the experimental period (irrigation) and the FW (Flores-Saavedra et al. 2025) using the formula: WUE = FW/Irrigation amount.

### Biochemical analyses

#### Photosynthetic pigments

The amounts of chlorophylls a (Chl_a) and b (Chl_b), and carotenoids (Caro) were determined according to the protocol of Lichtenthaler and Wellburn (1983). Fresh leaf material (0.05 g) was crushed and extracted in 1 mL of ice-cold 80% acetone to quantitatively assess photosynthetic pigments. The extraction was carried out overnight on a shaker in the dark. The samples were centrifuged at 13,300 × *g* for 15 min at 4 °C, and the supernatant was collected. The absorbance of the supernatant was measured at 470 nm, 646 nm, and 663 nm using a UV/visible spectrophotometer (UV-3100; VWR). The concentrations of chlorophylls a and b, and carotenoids were expressed in mg g^−1^ DW.

#### Osmolytes

Proline (Pro) and total soluble sugars (TSS) were analyzed. Pro was determined following the protocol of Bates et al. (1973). Fresh leaf material (0.05 g) was extracted with sulfosalicylic acid (3%, w/v) and mixed with acid ninhydrin, incubated in a water bath for 1 h at 98 °C, cooled on ice for 10 min, and extracted with toluene. The absorbance of the organic phase was measured at 520 nm. The standard curve was determined with solutions containing known Pro concentrations, assayed in parallel. Pro concentration was expressed as µmol g^−1^ DW.

TSS were measured according to Dubois et al. (1956). Fresh leaf samples were extracted overnight with 2 mL of methanol (80%, v/v). All samples were centrifuged at 15,500 × *g* for 10 min at 4 °C, and the supernatants were mixed with 500 µL of 5% phenol (v/v) and 2.5 mL of concentrated sulfuric acid. After a 20 min incubation at room temperature, the absorbance was measured at 490 nm. TSS contents were expressed as equivalents of glucose (mg eq. glucose g^−1^ DW).

#### Oxidative stress marker

The levels of MDA were measured in leaf extracts as described by Hodges et al. (1999). The 80% methanol extracts were mixed with 0.5% thiobarbituric acid (TBA) prepared in 20% trichloroacetic acid (TCA) or with 20% TCA without TBA for the control. Then, the samples were incubated in a dry block thermostat at 95 °C for 15 min and cooled on ice for 5 min to stop the reaction. After this step, the samples were centrifuged at 13,300 × *g* for 10 min at 4 °C. The absorbance of supernatants was measured at 400, 532, and 600 nm with a reference (blank) without TBA in plastic cuvettes. MDA concentrations were calculated as described by Hodges et al. (1999) and expressed as nmol g^−1^ DW.

### Data analysis

To assess the significance of differences observed for the traits evaluated across the eight accessions under control and WS conditions, an analysis of variance (ANOVA) was conducted using a general linear model. Pairwise comparisons were performed using the Student–Newman–Keuls (SNK) test at a significance level of p < 0.05. Analysis of variance and multiple comparison tests were performed using the statistical package program XLSTAT (v.2016.02.28451, Addinsoft, France). Principal component analysis (PCA) and correlations were conducted in R-Studio (R Core Team 2022). Pearson correlations were performed to determine relationships within each water treatment group (control and water stress) using the corrplot package (Wei and Simko 2021), whereas PCA was utilized to investigate relationships among the evaluated traits, accessions, and water stress treatment. All graphics were visualized using the ggplot2 package (Wickham et al. 2016).

## Results

### Morphological traits

**Fig. 1:**
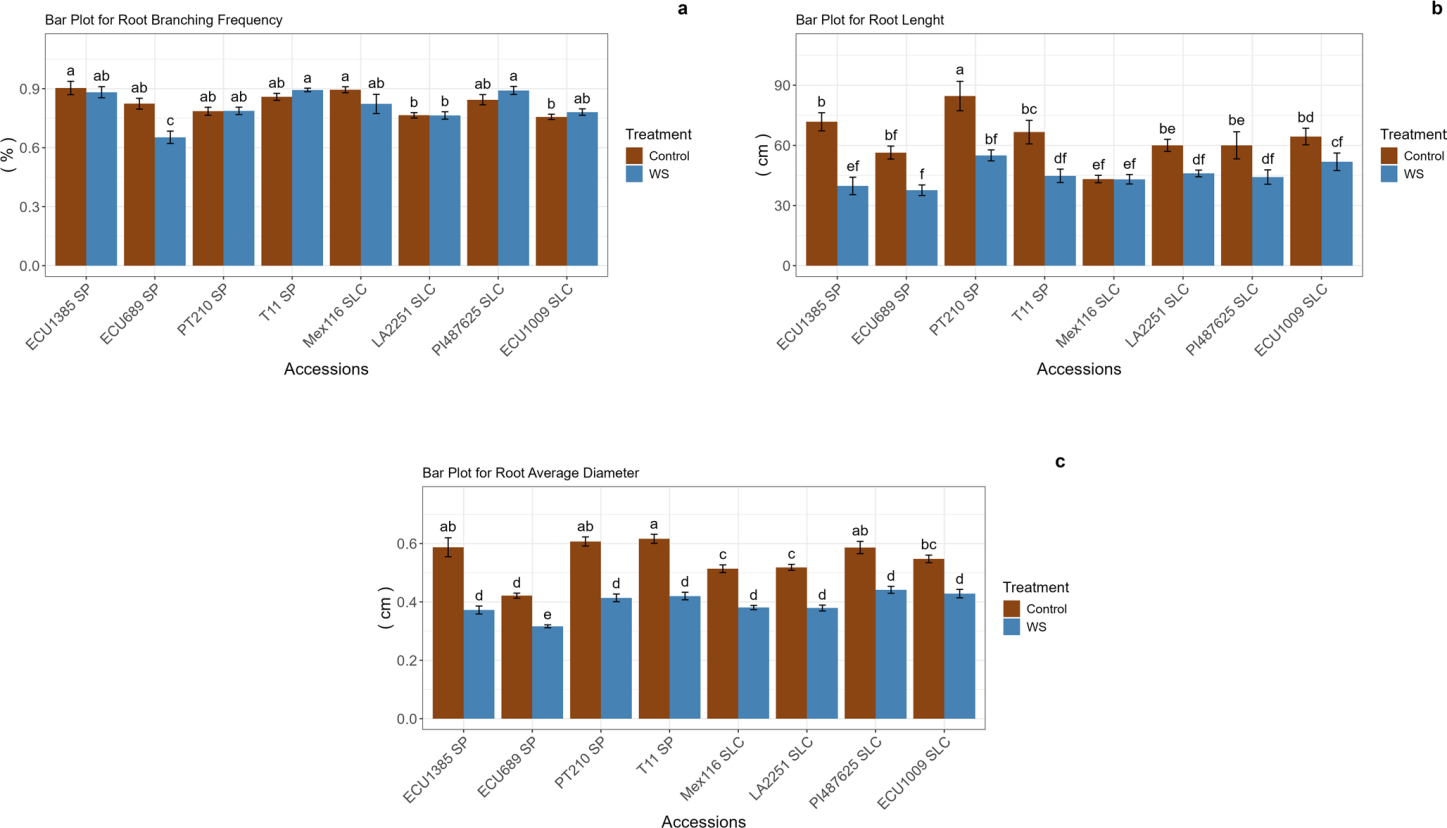
Root branching frequency (RBF; **a**), root length (RL; **b**) and root average diameter (RAD; **c**) values in *S. pimpinellifolium* (SP) and *S. lycopersicum* var. *cerasiforme* (SLC) accessions in the control and water stress treatments. Error bars represent the standard error. Means with different letters are significantly different according to the multiple range SNK test for p < 0.05

The irrigation treatment significantly influenced root traits, with notable differences observed among the eight accessions (Fig. 1). For RBF, no significant differences were detected between the control and water stress treatments in the accessions evaluated, except for ECU689, which experienced a 20.71% reduction due to water stress. The PT210 accession had the longest root length in the control group (84.6 cm). Under water stress conditions, root lengths were consistently lower across all accessions compared to the control. However, no significant statistical differences in root length were found between the control and WS treatments for the ECU689, Mex116, LA2251, PI487625 and ECU1009 accessions. In contrast, ECU1385, PT210 and T11 exhibited significant differences. For RAD, significant statistical differences were identified between full irrigation and WS treatments across all accessions. In all accessions, the RAD value in the control group was higher than that observed in the water stress treatment. Within the control group, T11 displayed the highest RAD value, while ECU1385, PT210 and PI487625 accessions were statistically similar to T11. The lowest RAD was recorded in the ECU689 accession. Under the water stress treatment, although the other accessions were statistically similar to one another, the RAD value of ECU689 was notably lower (Fig. 1).

**Fig. 2:**
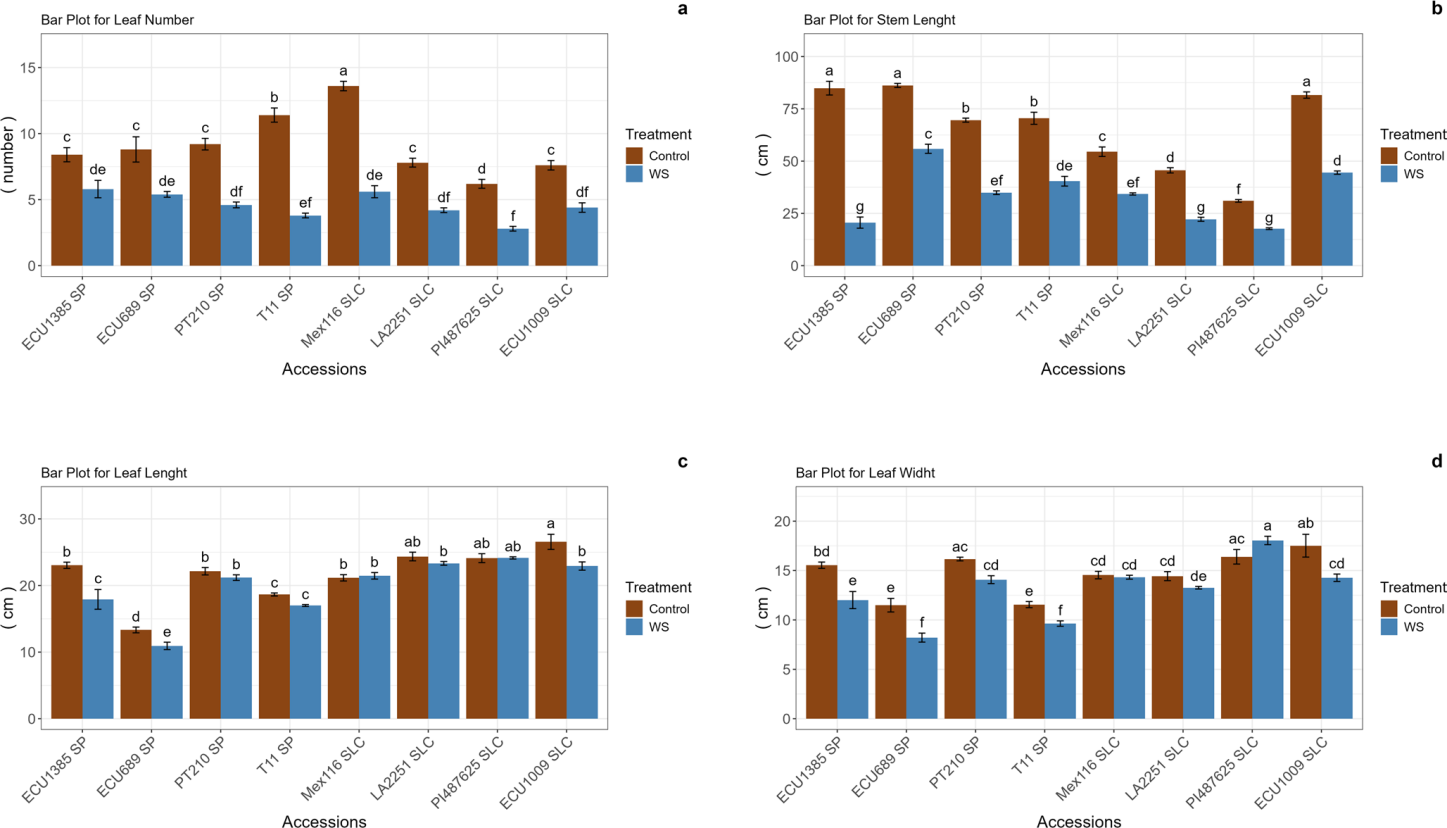
Leaf number (LN; **a**), stem length (SL; **b**), leaf length (LL, **c**), and leaf width (LW; **d**) values in *S. pimpinellifolium* (SP) and *S. lycopersicum* var. *cerasiforme* (SLC) accessions in the control and water stress treatments. Error bars represent the standard error. Means with different letters are significantly different according to the multiple range SNK test for p < 0.05

A significant difference was observed in aerial morphological traits between the control and water stress treatments for leaf number (LN) across all accessions (Fig. 2). Similarly, stem length (SL) exhibited statistically significant decreases in all accessions under water stress, indicating a consistent reduction in growth, with ECU1385 demonstrating the most pronounced decrease. Significant reductions in leaf length (LL), were noted in the ECU1385, ECU689, and ECU1009 accessions. For leaf width (LW), significant differences between the control and water stress treatments were identified in the ECU1385, ECU689, T11, and ECU1009 accessions. These findings indicate that water stress has substantial impact on stem growth and leaf number; however, its effects on leaf length and width vary depending on the specific accession (Fig. 2).

**Fig. 3:**
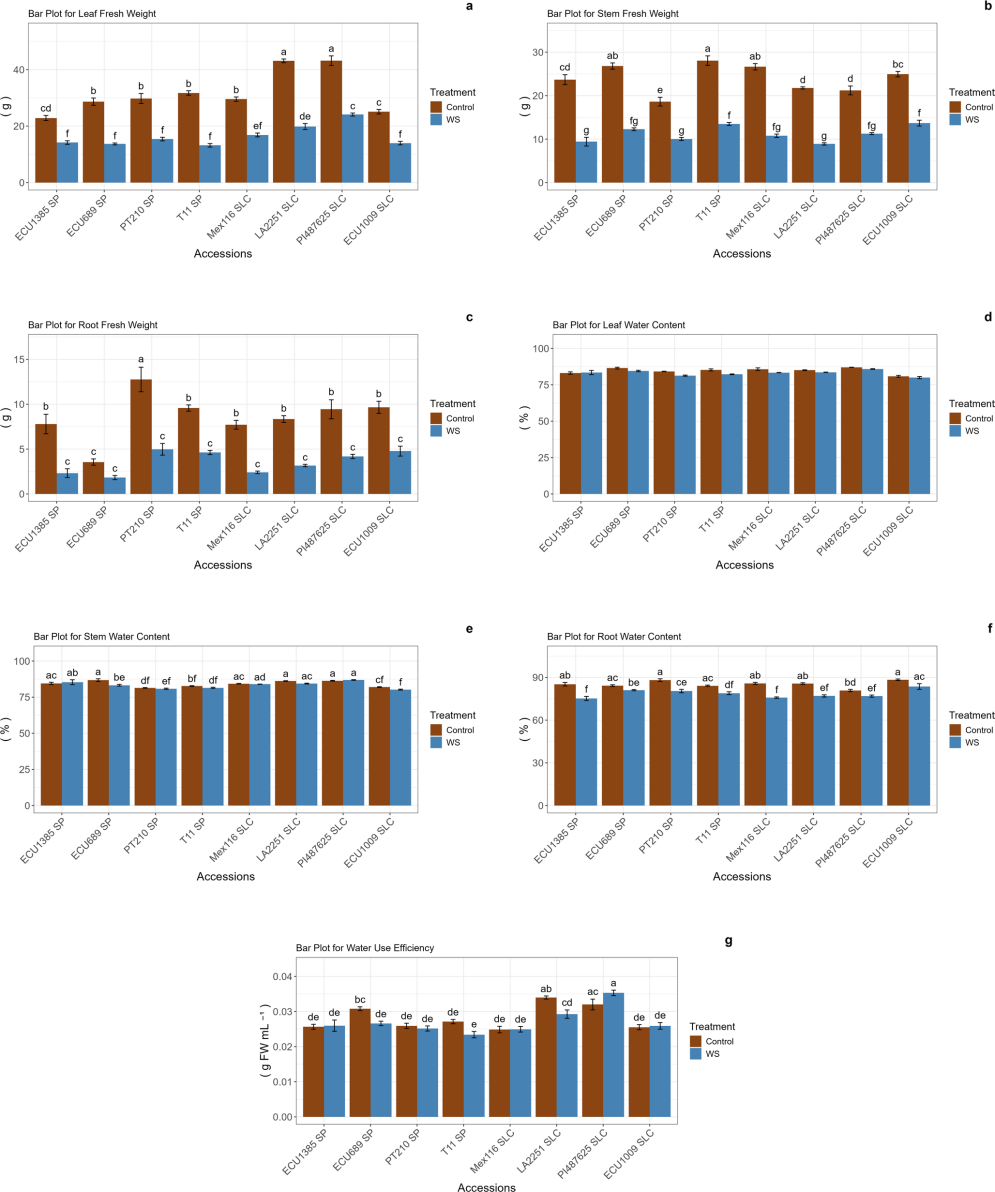
Leaf fresh weight (LFW; **a**), stem fresh weight (SFW; **b**), root fresh weight (RFW; **c**), leaf water content (LWC; **d**), stem water content (SWC; **e**), root water content (RWC; **f**), and water use efficiency (WUE; **g**) values in *S. pimpinellifolium* (SP) and *S. lycopersicum* var. *cerasiforme* (SLC) accessions in the control and water stress treatments. Error bars represent the standard error. Means with different letters are significantly different according to the multiple range SNK test for p < 0.05

Statistically significant differences were identified between the control and water stress treatments for leaf fresh weight (LFW) and shoot fresh weight (SFW) across all accessions (Fig. 3). For LFW, the most significant reduction between the control and water stress treatments was observed in the T11 and LA2251 accessions, which exhibited decreases of 58.36% and 53.97%, respectively. In contrast, the smallest reduction was recorded in ECU1385, which demonstrated a 38% decrease. Regarding SFW, ECU1385 displayed the most significant decline, decreasing from 23.68 g to 9.41 g, representing a 60.26% reduction. Conversely, ECU1009 experienced a decrease from 24.94 g to 13.68 g, amounting to a reduction of 45.15%. In terms of root fresh weight (RFW), statistically significant differences were observed in all accessions except for ECU689, indicating that RFW was not influenced by water stress conditions in this particular accession. With respect to leaf water content (LWC), no statistically significant differences were found among all accessions and treatments (Fig. 3). Moreover, while most accessions did not exhibit significant differences in soil water content (SWC) between the control and water stress treatments, ECU689 demonstrated a slight reduction from 86.80% to 83.25%. The ECU689 and ECU1009 accessions maintained relative water content (RWC), whereas other accessions showed significant variations between the control and water stress treatments. Finally, water use efficiency (WUE) differed significantly between the control and water stress conditions in ECU689 and LA2251, while no significant differences were observed between the control and water stress treatments in the remaining accessions (Fig. 3).

### Biochemical traits

**Fig. 4:**
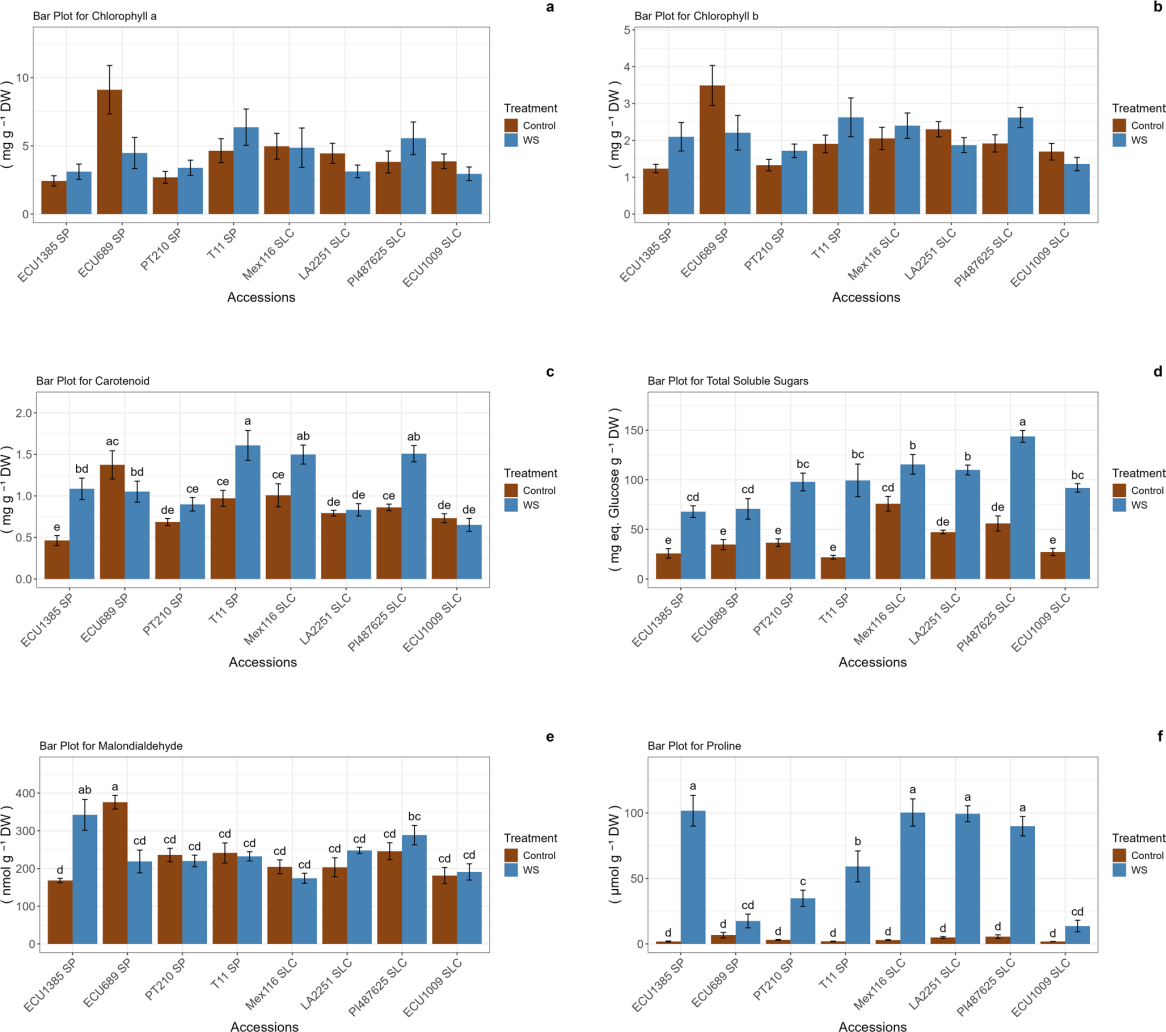
Chlorophyll a (Chl_a; **a**), Chlorophyll b (Chl_b; **b**), carotenoids (Caro; **c**), total soluble sugars (TSS; **d**), malondialdehyde (MDA; **e**), and proline (Pro; **f**) values in *S. pimpinellifolium* (SP) and *S. lycopersicum* var. *cerasiforme* (SLC) accessions in the control and water stress treatments. Error bars represent the standard error. Means with different letters are significantly different according to the multiple range SNK test for p < 0.05

In the present study, no statistically significant differences were observed in the concentrations of Chl_a and Chl_b between the control and water stress treatments across all accessions (Fig. 4). The accessions with the highest carotenoid content were identified as ECU689, T11, Mex116, and PI487625. Notably, while the majority of accessions exhibited peak carotenoid values under water stress, ECU689 displayed the highest value in the control group. Conversely, total soluble sugars (TSS) exhibited substantial increases under water stress, particularly in the PI487625 accession, where TSS values reached 143.76 mg eq. glucose g^−1^ DW under water stress, surpassing those of the control group (56.00 mg eq. glucose g^−1^ DW). Furthermore, the highest TSS was observed in the PI487625 accession. Malondialdehyde (MDA), a widely used marker for oxidative stress, demonstrated no statistically significant variation between treatments, with the exception of ECU1385 and ECU689. The highest MDA levels were recorded in the ECU689 accession within the control group, while elevated levels were detected in the ECU1385 accession under water stress conditions. Proline (Pro) levels exhibited significant increases under water stress across all accessions, with the exception of ECU689 and ECU1009. The most pronounced accumulation of proline was observed in the ECU1385, Mex116, LA2251, and PI487625 accessions, where proline levels reached 101.71, 100.39, 99.38, and 89.91 µmol g^−1^ DW, respectively, under stress conditions. In contrast, no significant differences were noted under control conditions across all accessions. Moreover, ECU689 and ECU1009 displayed proline levels of 17.52 and 13.66 µmol g^−1^ DW, respectively, under water stress, showing no statistically significant difference compared to the control group (Fig. 4).

**Fig. 5:**
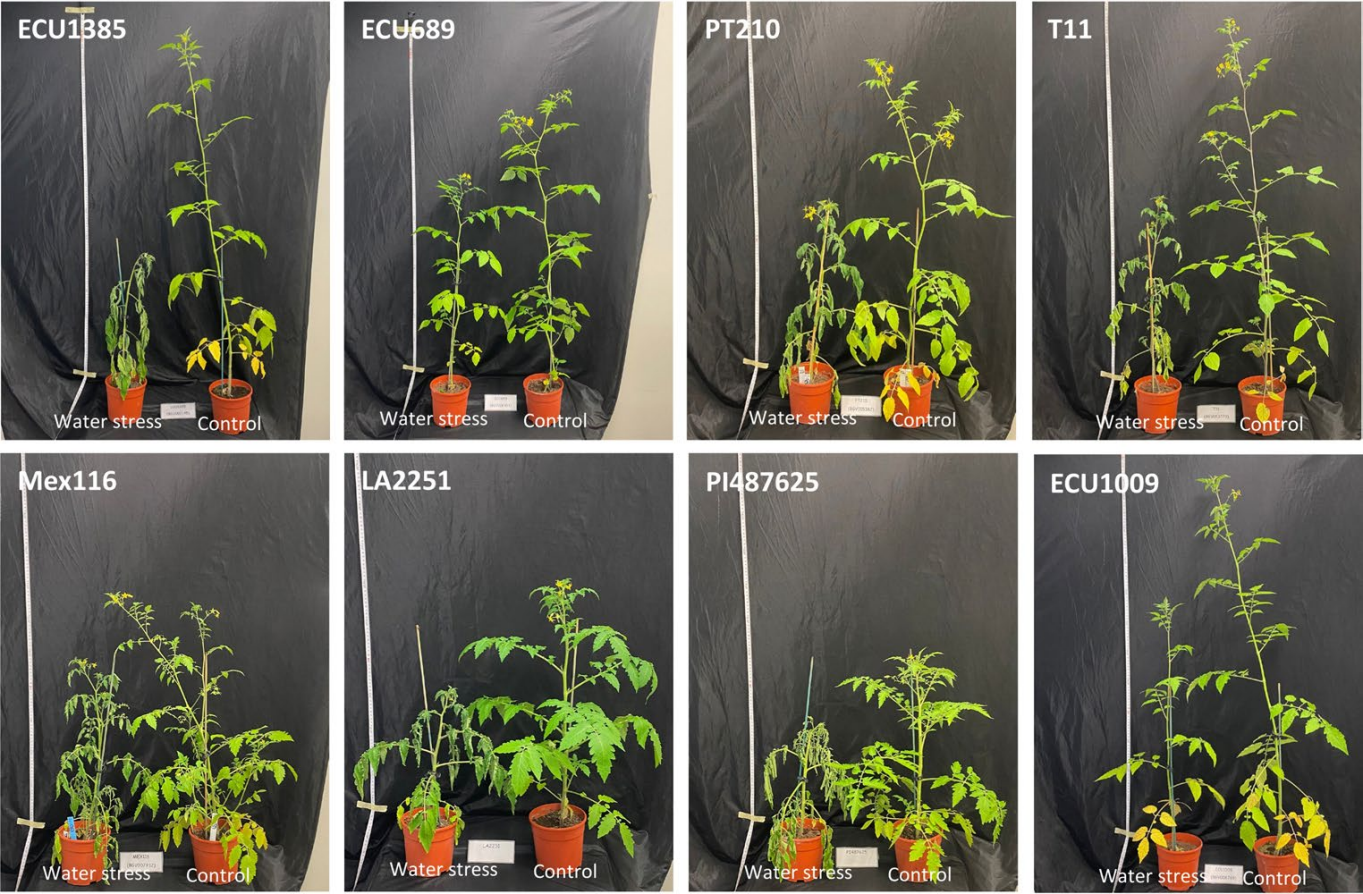
Water stressed and control plants in the *S. pimpinellifolium* and *S. lycopersicum* var. *cerasiforme* accessions evaluated

The effects of WS were also visually assessed on the eight accessions under control and water-stressed conditions at the end of the experiment (Fig. 5). The images clearly demonstrate that drought-tolerant accessions maintained overall plant development, including leaf structure and plant vigor, under water stress conditions. Notably, the ECU689 and ECU1009 accessions exhibited minimal morphological changes and less wilting symptoms, reflecting its strong adaptive mechanisms. In contrast, susceptible accessions such as PI487625 displayed visible reductions in overall plant growth, with clear signs of stress-induced developmental limitations.

### Correlation analysis

**Fig. 6:**
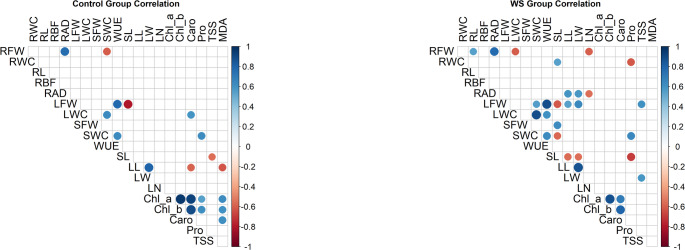
Pearson correlation matrix coefficients for *S. pimpinellifolium* (SP) and *S. lycopersicum* var. *cerasiforme* (SLC) accessions subjected to control (left) and water stress (right) treatments. The size of the circles represents the strength of the correlation, with larger circles indicating higher correlation values. Only significant correlations are shown (p < 0.05)

Several physiological and biochemical traits under control and water stress conditions showed significantly positive correlations (Fig. 6). Under control conditions, high positive correlations (r > 0.80) were observed between Chl_a, Chl_b and Caro. LFW positively correlated with WUE (r = 0.78) and negatively with SL (r = -0.81), whereas RFW showed a positive association with RAD (r = 0.76) but a negative correlation with SWC (r = -0.61). Additionally, SWC had a positive correlation with LWC (r = 0.64) and WUE (r = 0.63). In the control group, WUE is correlated with LFW and LWC. So, WUE is mostly related to the leaf. LL had a positive correlation with LW (r = 0.81). Another interesting result is that MDA had positive correlation with Chl_a, Chl_b and Caro (r = 0.62, 0.60 and 0.61, respectively) and negative correlation with LL (r = 0.62). Under water stress conditions, different patterns were observed. RFW was negatively correlated with LN and LWC (r = -0.60 and -0.60, respectively) and positively correlated with RAD (r = 0.78). Pro was positively correlated with SWC (r = 0.65), while a negative correlation was observed with SL and RWC (r = -0.69 and 0.61, respectively). SL also had a negative correlation with LFW and a positive correlation with SFW (r = -0.61 and 0.63, respectively). In addition, WUE positively correlated with LFW, LWC and SWC (r = 0.88, 0.61 and 0.64, respectively). Also, LWC negatively correlated with RFW (r = -0.60). SFW had a positive correlation with SL (r = 0.63). LL and LW had a significant positive correlation with each other (r = 0.87). Caro had a positive correlation with Chl_a and Chl_b, like the control group with the least significance (r = 0.69 and 0.80, respectively).

### Principal component analysis

**Fig. 7:**
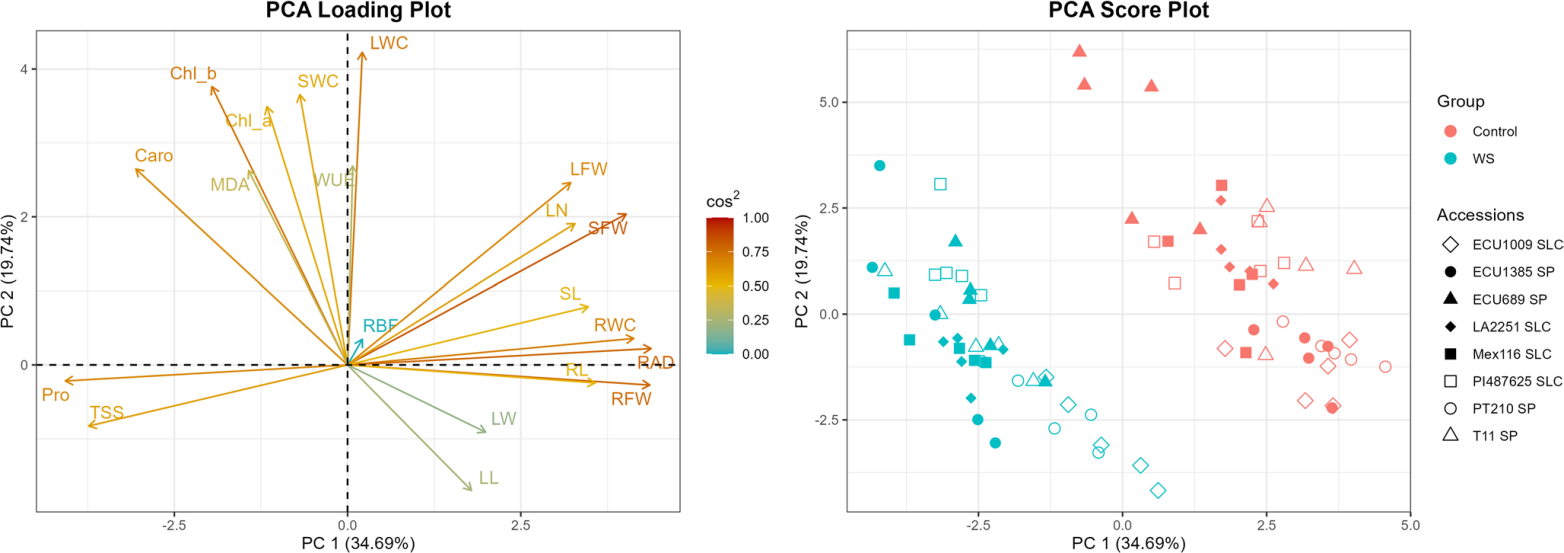
PCA loading plot (left) and score plot (right) of the 20 different traits for eight *S. pimpinellifolium* and *S. lycopersicum* var. *cerasiforme* accessions. The increasing arrow lengths and color tones, from blue to red, represent the contributions of the measured variables to the components. Solid markers in the score plot represent *S. pimpinellifolium* and open markers represent *S. lycopersicum* var. *cerasiforme*

The first principal component (PC1) accounted for 34.69% of the total variation, whereas the second one (PC2) explained 19.74%. The PCA loading plot reveals that PC1 is primarily driven by variables such as SFW, which is highly correlated with PC1, indicating its strong influence on the primary variability among tomato accessions, whereas other variables, like RBF, show very low correlations with PC1. The PCA score plot shows a clear separation of plants from the control and WS groups. In this way, samples under WS are generally positioned on the lower diagonal of the score plot, indicating an association with higher values of Pro and TSS, as seen in the loading plot. In contrast, control group samples cluster on the upper diagonal of the PCA plot, indicating an association with growth and water content traits. The distinct separation along PC1 highlights the significant impact of water stress on genotypic responses, with *S. pimpinellifolium* (filled shapes) and *S. lycopersicum* var. *cerasiforme* (hollow shapes) accessions showing a high degree of intermingling (Fig. 7).

## Discussion

Drought is one of the abiotic stress factors that threatens agriculture, inducing numerous physiological and biochemical alterations in plants that adversely impact growth and yield. In addition, global climate change further intensifies the frequency and severity of drought events (Anjum et al. 2011). Wild tomato species exhibit drought tolerance and represent significant genetic resources for breeding studies (Peralta et al. 2008). However, the understanding of trait combinations in these drought stress adapted species remains limited (Tapia et al. 2016). Understanding the morphological and biochemical responses of various tomato accessions to water stress is crucial for identifying traits correlated with drought tolerance. In this study, we assessed the responses of eight accessions of *S. pimpinellifolium* and *S. lycopersicum* var. *cerasiforme* to water stress, examining fundamental morphological traits such as root and shoot development, WUE, and physiological adaptations, including osmolyte accumulation and the stability of photosynthetic pigments. Our findings provide valuable insights into the drought tolerance exhibited by various accessions. Notably, the *S. pimpinellifolium* ECU689 and *S. lycopersicum* var. *cerasiforme* ECU1009 accessions demonstrated tolerance to water stress, highlighting their potential as promising candidates for breeding programs. Also, PCA and correlation analyses were used as exploratory tools to outline the multivariate structure of parental trait variation, rather than to test specific breeding hypotheses. This approach allows the identification of stress-responsive traits that will guide future breeding-oriented analyses in the MAGIC population.

It should be noted that this study was conducted under controlled greenhouse conditions, which enabled precise management of the water deficit and minimized environmental variability (Flores-Saavedra et al. 2023). Although such controlled settings are ideal for isolating genotypic responses, they do not fully capture the complexity of field environments. Because most variables analyzed here are mechanistic (e.g., pigments, osmolytes, oxidative markers) and yield was not measured, drought tolerance indices, whose interpretation is most meaningful for agronomic targets (Negisho et al. 2022), were not computed. Instead, we focused on integrative morphological and biochemical parameters that collectively describe the plant’s response to water deficit (Plazas et al. 2022), including WUE, RWC, photosynthetic pigment stability, osmolyte accumulation, and oxidative stress markers. While additional physiological variables such as stomatal conductance, leaf water potential, and root-to-shoot ratio could provide further insight (Parkash and Singh 2020; Seleiman et al. 2021), these were not included because the experiment aimed to capture stable mechanistic responses rather than short-term gas-exchange dynamics. It is important to note that the 30-day duration of the water stress treatment represents a limitation, as it reflects short-term mechanistic responses rather than long-term acclimation. Consequently, the results reported here should be interpreted as short-term adaptive responses under controlled conditions. Future multi-cycle and field evaluations of these accessions and the derived MAGIC population will incorporate several tolerance indices and additional physiological measurements to complement the mechanistic evidence reported here and validate drought responses under agronomic conditions.

Roots play a critical role in the plant's ability to adapt to stress, influencing overall performance (Lynch 1995), and tomato root traits such as root length, diameter, and volume are crucial indicators of a plant’s ability to withstand stress conditions (Suchoff et al. 2017). Studies on the effect of drought stress on root development in tomatoes have shown that stress particularly promotes root length, with tomato plants prioritizing the elongation of the primary root to access deeper soil layers where water may be more available (Machado et al. 2022). Increased root length has also been reported to enhance water acquisition (Comas et al. 2013). Furthermore, data from studies conducted on different soils indicate that drought stress strongly stimulates deeper root development, especially in sandy soils (Alaoui et al. 2014). In our study, no significant differences were observed for root branching frequency between the control and water stress treatments, except for ECU689. In contrast, in this accession no differences were found in root length (RL), root fresh weight (RFW), and relative water content (RWC), which are critical for water uptake and retention under drought conditions. These findings highlight the differential responses of root traits under water stress.

Tomato plants exhibit significant variation in morphological traits, particularly under drought stress conditions. Traits such as LN, SL, LL, and LW are crucial indicators of plant performance in water-limited environments (Parveen et al. 2019). Many studies have determined that these morphological characteristics differ among tomatoes (Tapia et al. 2016). Martínez-Cuenca et al. (2020) previously identified accessions such as ECU689 and PT210 lines as tolerant to water deficit and salt stress, and our results further support this classification. In our study, significant differences were observed between the control and stress treatments regarding leaf number (LN) and stem length (SL) across all accessions. Notably, a decrease in leaf length (LL) was recorded in the ECU1385, ECU689, and ECU1009 accessions, while reduction in leaf width (LW) was noted in the T11, in addition to the previously mentioned accessions. The ability to maintain morphological traits, such as leaf and stem parameters, under drought stress has been reported as a critical feature of stress-tolerant tomato genotypes (Helyes et al. 2012; Zegbe et al. 2003).

Statistically significant differences were observed between the control and water stress treatments regarding LFW and SFW across all accessions. However, for RFW, differences between treatments were found in all accessions except for ECU689. The findings reported by Zhou et al. (2017) and Raja et al. (2020) indicate a significant reduction in these traits in tomato plants subjected to drought stress conditions. Water stress had varied effects on the relative water content (%) of leaves, stems, and roots in the tomato accessions. No significant differences were found in LWC in all accession. Under stress conditions, plants maintain their physiological balance through higher RWC values (Raja et al. 2020). Several physiological traits are linked to water availability, and most studies have demonstrated that high RWC is closely related to drought resistance (Maghsoudi et al. 2016; Sánchez-Rodríguez et al. 2010; Rosales et al. 2013; Zegaoui et al. 2017). The observed variation in WUE among the accessions ECU689 and LA2251 under control and water stress conditions highlights the differential physiological responses of these accessions to drought stress. These results suggest that water stress affects the ability of these accessions to optimize water use, potentially reflecting differences in how each accession copes with limited water availability. The variation in WUE responses across different accessions highlights the importance of considering both genetic diversity and environmental factors when assessing drought tolerance mechanisms in tomato plants. Our study discovered significant differences in WUE and demonstrated the ability to cope with water stress by maintaining stable physiological traits such as RWC. The relative stability of ECU689 and ECU1009 in terms of root water content (RWC) suggests that these accessions have developed an effective defence mechanism in the root zone against water stress. These traits indicate that ECU689 and ECU1009 exhibit greater resilience to water stress and may utilize water more efficiently. These accessions likely maintain the physical integrity of their root systems and may develop deeper roots, enabling better adaptation to water stress. Although root length, diameter, and water content were quantified, we did not measure root depth or water extraction along the soil profile; therefore, any reference to deeper rooting behaviour reflects functional interpretation of the observed stability of root traits under stress rather than direct measurement. These findings demonstrate that ECU689 and ECU1009 maintain root system stability under water stress, showcasing a more robust profile compared to other accessions. In this way, the contrasting responses observed among the parental lines, especially the stable root water content and osmotic regulation in ECU689 and ECU1009, define valuable trait combinations that can be targeted for introgression and later exploited through marker-assisted approaches in the derived MAGIC population.

The photosynthetic pigments chlorophylls and carotenoids provide essential functions in light harvesting and photoprotection of the photosynthetic tools (Simkin et al. 2022), and they serve as an indicator of chloroplast development and photosynthetic performance. Drought stress is a critical factor that causes a decrease in chlorophyll content and reduces the plant’s photosynthetic efficiency (Munné-Bosch and Alegre 2000) and this stress can also affect carotenoid accumulation; these compounds protect against oxidative stress by preventing chlorophyll damage (Havaux 1998). However, in the present study, no significant differences were observed between the control and stress treatments for these traits across all accessions. Certain genotypes may exhibit intrinsic mechanisms that protect the photosynthetic apparatus, even under stress conditions. There were significant differences in carotenoid concentration among the accessions utilized in the study. The concentration of all carotenoids increased under water stress conditions, with ECU689 exhibiting the highest value compared to the control group. Moreover, the correlation between drought stress and modifications in pigment composition can display considerable variability, depending on the specific plant species and genotype under consideration. (Anjum et al. 2003).

The rise in TSS levels under stress could be attributed to the osmotic adjustment strategy that plants use to adapt to water stress, with numerous studies indicating that this increase varies across different cultivars (Helyes et al. 2012; Zegbe et al. 2003). Proline is essential for osmoprotection, osmotic adjustment, and redox regulation in many plant species. Plants accumulate free Pro in response to abiotic stress (Hong et al. 2000). Several studies have shown that tomato plants under stress exhibit higher Pro levels compared to controls (Ghorbanli et al. 2013; Mohamadi and Rajaei 2013; Murtaza et al. 2024; Rai et al. 2006; Tal et al. 1979). Also, Pro accumulation was found to be higher in stress-tolerant tomato plants than in stress-sensitive ones when subjected to drought and salinity treatments (Patanè et al. 2022; Kahlaoui et al. 2014). However, other studies on tomato (Aziz et al. 1998; Tal et al. 1979*)* reported the opposite trend, showing a negative correlation between salt tolerance and proline accumulation. These studies reveal that the association between proline accumulation and drought tolerance is not universal, highlighting that the functional relevance of proline is highly genotype- and context-dependent. In our case, the lack of a difference in proline levels between the water deficit and control treatments in ECU689 and ECU1009 suggests that these accessions may rely less on proline accumulation and more on other stress response mechanisms, such as the synthesis of alternative osmolytes, regulation of ion transport, or activation of antioxidant systems, with the relative importance of these responses varying between genotypes. The reduced proline levels observed in ECU689 and ECU1009 under water stress conditions indicate that these genotypes experience diminished stress compared to others. Consequently, it demonstrates a higher tolerance, thereby supporting the hypothesis that proline functions as a biomarker for stress. Unlike other accessions, the response of ECU689 and ECU1009 indicates a potential physiological adaptation to stress conditions.

In addition, in some genotypes, proline concentrations may be high enough to contribute to osmotic adjustment, while in others, these levels may be too low, indicating that proline's capacity to provide osmotic effect could be limited in some accessions. Other accessions exhibited a more pronounced increase in proline levels under stress conditions, suggesting a greater dependence on proline for osmotic adjustment and protection against dehydration.

These results underscore the importance of biochemical diversity in drought tolerance strategies among tomato accessions.

## Conclusion

Drought is the most important abiotic stress factor negatively affecting tomato growth and development (Liang et al. 2020). In the present study, the response of *S. pimpinellifolium* and *S. lycopersicum* var. *cerasiforme* accessions to water stress was investigated by determining physiological, morphological, and biochemical parameters. The results show that water stress significantly alters critical traits such as root morphology, photosynthetic pigments and osmolyte accumulation. Among the accessions used, ECU689 (SP) and ECU1009 (SLC) exhibited superior tolerance under water stress conditions with physiological traits such as constant proline levels. Our results reveal that these accessions have developed or utilized various adaptation responses to cope with extreme environmental factors such as drought and are excellent candidates for breeding drought-tolerant tomato varieties. Proline, chlorophylls, and carotenoids are examples of biochemical markers that have become important indicators of drought responses. In particular, proline showed a strong positive correlation with osmotic balance under water stress and plays an important role in maintaining cellular hydration and metabolic activity. This study significantly contributes to the understanding of drought tolerance in tomato close relatives and provides a sound basis for developing stress-tolerant varieties. These findings indicate that the ECU689 and ECU1009 accessions exhibit enhanced drought tolerance and, in consequence, are of great interest for being incorporated in tomato breeding pipelines aiming at developing drought tolerant cultivars or rootstocks.

## Supplementary Information

Below is the link to the electronic supplementary material.

Supplementary Material 1

## Data Availability

Data will be made available on request.
