# Supplementary material for: Morphological and biochemical responses to water stress in Solanum pimpinellifolium and S. lycopersicum var. cerasiforme accessions
Source: Physiol Mol Biol Plants. 2026 Jan 20;32(1):149–62. doi: 10.1007/s12298-026-01705-7 (PMC12886681; doi:10.1007/s12298-026-01705-7)
Supplement: Supplementary file 1 — Supplementary Material 1 [file 12298_2026_1705_MOESM1_ESM.docx]

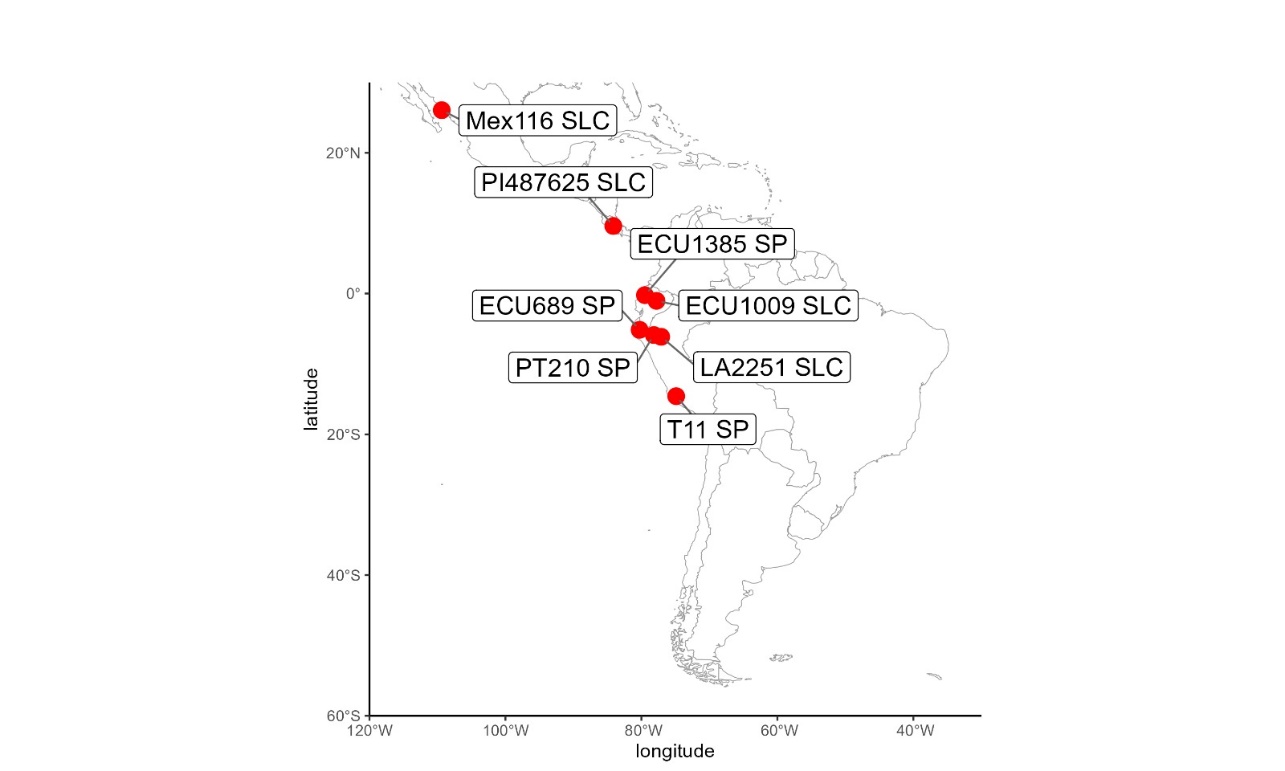


Fig. S1. Geographical origin of four *S. pimpinellifolium* (SP) and four *S. lycopersicum* var. *cerasiforme* (SLC) accessions evaluated for tolerance to drought (modified from Arrones et al., 2024).
